# Relationships among Inflammatory Biomarkers and Self-Reported Treatment-Related Symptoms in Patients Treated with Chemotherapy for Gynecologic Cancer: A Controlled Comparison

**DOI:** 10.3390/cancers15133407

**Published:** 2023-06-29

**Authors:** Aasha I. Hoogland, Brent J. Small, Laura B. Oswald, Crystal Bryant, Yvelise Rodriguez, Brian D. Gonzalez, Xiaoyin Li, Michelle C. Janelsins, Hailey W. Bulls, Brian W. James, Bianca Arboleda, Claudia Colon-Echevarria, Mary K. Townsend, Shelley S. Tworoger, Paulo C. Rodriguez, Julienne E. Bower, Sachin M. Apte, Robert M. Wenham, Heather S. L. Jim

**Affiliations:** 1 Department of Health Outcomes and Behavior, Moffitt Cancer Center, Tampa, FL 33612, USA; aasha.hoogland@moffitt.org (A.I.H.);; 2 School of Aging Studies, University of South Florida, Tampa, FL 33612, USA; 3 Department of Surgery and Neuroscience, University of Rochester Medical Center, Rochester, NY 14642, USA; 4 Department of Medicine, University of Pittsburgh, Pittsburgh, PA 15213, USA; 5 Morsani College of Medicine, University of South Florida, Tampa, FL 33602, USA; 6 Department of Cancer Epidemiology, Moffitt Cancer Center, Tampa, FL 33612, USA; 7 Department of Immunology, Moffitt Cancer Center, Tampa, FL 33612, USA; 8 Department of Psychology, University of California Los Angeles, Los Angeles, CA 90095, USA; 9 Department of Obstetrics and Gynecology, Huntsman Cancer Institute, Salt Lake City, UT 84132, USA; 10 Department of Gynecologic Oncology, Moffitt Cancer Center, Tampa, FL 33612, USA

**Keywords:** chemotherapy, cytokines, gynecologic cancer, quality of life in cancer patients

## Abstract

**Simple Summary:**

Treatment-related symptoms such as fatigue, depression, and disruptions in sleep and physical activity are common and distressing in gynecologic cancer patients. The aim of our study was to examine whether higher levels of inflammation are associated with worse symptomatology, and if these associations are stronger for patients with gynecologic cancer (*n* = 121) than age-matched women without a cancer history (i.e., controls; *n* = 105). Elevated levels of C-reactive protein were associated with depression and disrupted physical activity, but there were no other significant associations between inflammation and treatment-related symptoms. Findings suggest that inflammation may not play a significant role in the development of fatigue or sleep disturbance among gynecologic cancer patients but may contribute to depression and physical inactivity.

**Abstract:**

Previous research suggests that inflammation triggers cancer-treatment-related symptoms (i.e., fatigue, depression, and disruptions in sleep and physical activity), but evidence is mixed. This study examined relationships between inflammatory biomarkers and symptoms in patients with gynecologic cancer compared to age-matched women with no cancer history (i.e., controls). Patients (*n* = 121) completed assessments before chemotherapy cycles 1, 3, and 6, and 6 and 12 months later. Controls (*n* = 105) completed assessments at similar timepoints. Changes in inflammation and symptomatology were evaluated using random-effects mixed models, and cross-sectional differences between patients and controls in inflammatory biomarkers and symptoms were evaluated using least squares means. Associations among inflammatory biomarkers and symptoms were evaluated using random-effects fluctuation mixed models. The results indicated that compared to controls, patients typically have higher inflammatory biomarkers (i.e., TNF-alpha, TNFR1, TNFR2, CRP, IL-1ra) and worse fatigue, depression, and sleep (*p*s < 0.05). Patients reported lower levels of baseline physical activity (*p* = 0.02) that became more similar to controls over time. Significant associations were observed between CRP, depression, and physical activity (*p*s < 0.05), but not between inflammation and other symptoms. The results suggest that inflammation may not play a significant role in fatigue or sleep disturbance among gynecologic cancer patients but may contribute to depression and physical inactivity.

## 1. Introduction

Fatigue, depression, and disruptions in sleep and physical activity, known collectively as treatment-related symptoms, tend to be common and distressing in gynecologic cancer patients [1,2,3,4,5], due in part to the arduous platinum- and taxane-based chemotherapy regimens prescribed as first- and second-line treatments [6]. Previous research has shown that symptoms occur in a cascade pattern during chemotherapy to treat gynecologic cancer, with disrupted sleep contributing to next-day increases in fatigue, and fatigue contributing to next-day increases in depression [7,8]. Interventions that target treatment-related symptoms early in the cascade may mitigate later symptoms, but the underlying biology that drives this cascade is yet to be elucidated.

There is strong evidence for a causal role of inflammation in symptomatology outside the context of cancer. Therapeutic and experimental administration of pro-inflammatory cytokines induces fatigue, depression, and altered sleep and activity patterns in both humans and animals [9,10,11,12,13,14,15,16,17,18,19,20,21]. In addition, inflammatory biomarkers are known to activate inflammatory processes in the brain, which in turn regulate neurotransmitters involved in mood and behavior, such as serotonin, norepinephrine, and dopamine [22,23,24,25,26]. However, the existing literature examining the relationship between inflammatory biomarkers and symptomatology in cancer patients is mixed and interpretation is limited by methodological challenges [27].

Previous studies of the relationships between inflammatory biomarkers and symptomatology in cancer have typically been characterized by cross-sectional study designs; a lack of non-cancer comparison groups to facilitate the interpretation of results; and patients that are heterogeneous in terms of cancer type, treatment received, and time since treatment completion. The majority of work has examined cancer survivors several months or years after the completion of treatment [27], with few studies examining inflammatory biomarkers and symptomatology both during and shortly after treatment [28,29,30]. Cross-sectional studies have found that post-treatment breast cancer survivors who report high levels of fatigue and depression also demonstrate higher levels of interleukin 1 receptor antagonist (sIL-1ra), interleukin 6 (IL-6), soluble IL-6 receptor (sIL-6r), soluble tumor necrosis factor receptor type II (sTNFR-II), and C-reactive protein (CRP) compared to those with low levels of symptomatology [31,32,33], although other studies of multiple cancer types have found no association [34,35,36]. In some studies of breast cancer survivors, cross-sectional associations have been found between inflammation and fatigue, but not depression or sleep problems [37]. Research examining these relationships over time has found that treatment-related increases in IL-6, sTNF-R1, and CRP are associated with worsening fatigue in patients with multiple cancer types [28,30,38]. Further, studies of breast cancer patients in the first 18 months after completing treatment suggest that psychological risk factors such as perceived stress may moderate the relationship between inflammation (i.e., CRP in one study, and the combination of CRP, IL-6, and sTNF-RII in another study) and depression [39,40]. Collectively, these findings raise the question of whether changes in inflammation levels affect symptomatology.

According to the 3P Factors model [41], inflammation may be a predisposing factor that increases the likelihood of worse symptomatology, a precipitating factor that hastens the onset of symptomatology, and/or a perpetuating factor that worsens or prolongs symptoms. For example, research on inflammatory biomarkers in cancer patients suggests that inflammation is altered prior to starting adjuvant chemotherapy, by the cancer itself and/or surgery. Advanced ovarian cancer is associated with greater circulating pro-inflammatory cytokines before surgery than early-stage disease [42]. Surgery is also known to trigger inflammatory processes [43]. In addition, high levels of symptom severity have been observed prior to the start of chemotherapy [44,45]. Research on breast cancer patients before treatment with radiotherapy or chemotherapy has also shown that fatigue is associated with higher circulating levels of pro-inflammatory cytokines [46].

Some studies suggest that inflammatory biomarkers are triggered by chemotherapy. In preclinical studies, platinum- and taxane-based chemotherapy elicit expression of interleukin 1 beta (IL-1β), IL-6, interleukin-8 (IL-8), interleukin-12 (IL-12), and tumor necrosis factor alpha (TNF-α) in primary human monocytes, macrophages, plasma, and various breast and ovarian cancer cell lines [47,48,49,50,51,52]. Interestingly however, there are few data regarding the effects of chemotherapy on circulating pro-inflammatory cytokines in humans [53,54,55,56]. There are even fewer data examining the relationship between pro-inflammatory cytokines and symptoms during chemotherapy; thus, it is unclear if inflammation precipitates symptoms. In patients with breast cancer, IL-8 was associated with flulike symptoms measured over three occasions during the first chemotherapy cycle [51], and soluble intercellular adhesion molecule 1 (sICAM-1) was associated with sleepiness before the first and fourth chemotherapy infusions [57]. In patients receiving chemoradiation for non-small cell lung cancer (NSCLC), advanced colorectal cancer, or advanced esophageal cancer, increases in sTNF-R1 and serum IL-6 have been associated with increases in fatigue and sleep disturbance [30,58]. However, other research in lung cancer patients undergoing chemotherapy found no association between IL-6, IL-8, or CRP with symptoms, including fatigue, sleep disturbance, and depression [59].

There is additional research in cancer patients suggesting that inflammatory biomarkers are triggered after chemotherapy is complete (i.e., late effects of treatment); data indicate that symptoms are worse in post-treatment cancer patients than individuals without cancer [60,61,62]. While it was previously assumed that these differences reflected persistent, acute effects of treatment, research by our group and others indicates that chemotherapy may be associated with late-onset symptoms not present at the end of treatment [63,64,65,66]. Thus, it may be that inflammation perpetuates symptoms during or after treatment. These observations are intriguing and merit additional investigation.

The goal of this study was to examine longitudinal relationships among changes in circulating pro- and anti-inflammatory biomarkers (i.e., IL-10, IL-1b, TNF-alpha, IL-6, IL-1ra, TNFR1, TNFR2, and CRP) and patient-reported symptoms in women with gynecologic cancer throughout the chemotherapy treatment trajectory. Specifically, our aim was to examine whether higher levels of inflammation were associated with worse symptomatology, and if these associations were stronger for patients than age-matched women without a cancer history (i.e., controls). Because inflammatory biomarkers can fluctuate very rapidly and individual measurements of inflammatory biomarkers may not reflect systemic or stable levels of inflammation, our analytic focus was on examining inflammation relative to participants’ personal average levels of inflammation, and relative to other participants’ average levels of inflammation. It was hypothesized that (1) patients would demonstrate higher levels of inflammation and worse symptomatology than controls throughout the treatment trajectory, (2) participants with higher levels of inflammation would report worse symptomatology than those with lower levels of inflammation (i.e., between-person effects), and (3) at times when participants had higher levels of inflammation than their own average, they would also report worse symptomatology than usual for them (i.e., within-person effects). We also explored whether there were differences in relationships among circulating inflammatory biomarkers and patient-reported symptomatology in patients vs. controls. We previously reported on symptoms among patients during active treatment [67]. For this paper, we expand on those findings to report on symptoms over one year post-treatment.

## 2. Materials and Methods

### 2.1. Participants

Study participants were recruited as part of a larger study examining the side effects of chemotherapy in patients with gynecologic cancer. As described previously [67], 6935 patients were screened for participation, 264 were approached, and 150/264 (57%) provided consent. Control participants were recruited from women identified through a national marketing company. A total of 355 controls were screened for participation, 346 were approached, and 150/346 (43%) provided consent. A sample size of 150 per group was chosen in order to have 80% power to detect between-group differences in slopes of at least 20% [68], assuming an alpha of 0.05 (two-tailed), intra-class correlation (ICC) of 0.50, and eight measurement occasions (in the larger study, data were collected before and after the first, third, and sixth infusion, and 6 and 12 months after completing chemotherapy). Eligible participants for both patient and control groups were (a) 18–89 years of age, (b) without psychiatric or neurological disorders that could interfere with study participation (e.g., dementia, psychosis), (c) without reported or documented diagnosis of an immune-related disease (e.g., HIV, systemic lupus erythematosus, rheumatoid arthritis), (d) not pregnant, (e) able to speak and read English, and (f) able to provide informed consent. Additional eligibility criteria for patients were (g) diagnosed with gynecologic cancer (i.e., ovarian, endometrial, peritoneal, fallopian, cervical, uterine, or vaginal), and (h) scheduled to receive intravenous or intraperitoneal chemotherapy at Moffitt Cancer Center (Tampa, FL, USA). Additional eligibility criteria for non-cancer controls were (i) not diagnosed with any form of cancer (except non-melanoma skin cancer), (j) age within five years of the patient participant to whom they were being matched, and (k) having a mailing address, working telephone number, and internet access. Participants were recruited between August 2013 and October 2018. This study was approved by the University of South Florida Institutional Review Board (Pro00005797).

Potential patient participants were identified by the research team in collaboration with their treating physician and were contacted via phone or in person to determine initial eligibility and interest in the study. Eligible and interested patients provided written informed consent to participate. Patient participants completed assessments before their first, third (i.e., approximately middle), and sixth (i.e., approximately last) chemotherapy infusions, and 6 and 12 months after their sixth chemotherapy infusion. Control participants provided written informed consent and completed assessments at equivalent time points.

### 2.2. Measures

#### 2.2.1. Demographic and Clinical Data

Patients completed a baseline questionnaire that assessed sociodemographic characteristics prior to starting chemotherapy and controls completed at time of enrollment, including date of birth, race, ethnicity, marital status, education level, household income, and menopausal status. Medical comorbidities were ascertained via a self-report version [69] of the Charlson Comorbidity Index [70]. Clinical characteristics for gynecologic cancer patients were obtained by a medical record review at baseline and included cancer type, stage, and previous chemotherapy.

#### 2.2.2. Circulating Inflammatory Biomarkers

Participants provided blood samples at baseline (i.e., before starting chemotherapy for patients), before chemo cycles 3 and 6, and again at 6 and 12 months after completing chemotherapy. Each serum or plasma blood sample was evaluated for the presence of inflammatory biomarkers, including IL-10, IL-1beta, TNF-alpha, TNFR1, TNFR2, CRP, IL-6, and IL-1ra. These inflammatory biomarkers were selected because they have shown significant associations with symptomatology in previous research and are readily detectable using existing laboratory methodology [31,32,33,71,72,73]. Blood samples were typically drawn at the same time of day (i.e., 8:00 a.m.–12:00 p.m.). Participants were asked to refrain from exercise, alcohol consumption, caffeine use, and non-prescription medications for the 24 h prior to blood draws [74,75]. Blood samples were sent to the Cancer Control and Psychoneuroimmunology Lab at the University of Rochester for analysis. All samples were assayed in one run using a multiplexed cytokine bead assay (i.e., IL-10, IL-1beta, TNF-alpha [HSTCMAG-28SK-04], TNFR1, TNFR2 [HSCRMAG-32K-02], IL-6, and IL-1ra [HCYTOMAG-60K-01]) or enzyme-linked immunosorbent assays (i.e., CRP; R&D Systems Human Quantikine ELISA; Minneapolis, MN, DCRP00) per the manufacturer’s instructions. All kits were from the same lot. For Luminex, the median concentration was taken from 50 beads per well. For ELISA, the average was taken from duplicates. All data and internal controls were inspected for a CV < 20%, with all kits run with a standard curve with an r^2^ > 0.98. All sample collections from the same participant were run on the same plate. The lower limits of detection of the assays, with sample dilution taken into account, were IL-10 = 0.30; IL-1β = 0.14; IL-6 = 0.04; TNFα = 0.08; IL-1Rα = 7.41; TNFR-1 = 10.60; TNFR-2 = 10.18; CRP = 5.0 pg/mL.

#### 2.2.3. Treatment-Related Symptoms

Fatigue was measured using the Fatigue Symptom Inventory [76]. The average of 4 items assessing the highest, lowest, average, and current levels of fatigue over the previous week was used in analyses. Scores ranged from 0 to 10 with higher scores indicating greater fatigue. A score of 3 and above indicated clinically meaningful fatigue [77].

Depression was assessed using the 7-item depression subscale of the Hospital Anxiety and Depression Scale designed to detect depressive symptoms in medically ill patients, including people with cancer [78,79]. Participants rated each item on a 4-point scale from 0 (absence) to 3 (extreme presence). All items were summed with possible scores ranging from 0 to 21. Scores of 8 or higher indicated clinically meaningful depressive symptoms [80].

Sleep was evaluated using the 19-item Pittsburgh Sleep Quality Index (PSQI) that assesses types and frequency of sleep disturbance experienced over the last month. All items were summed to derive an overall sleep quality score ranging from 0 to 21. Scores of 5 or higher indicated clinically meaningful sleep difficulties [81].

Physical activity was measured using the International Physical Activity Questionnaire-Short Form (IPAQ). The IPAQ assesses the frequency (days per week) and duration (minutes per day) of physical activity in the last 7 days [82]. Values were weighted by energy requirements for activities of varying intensities, which were defined by metabolic equivalents (METs) as follows: walking = 3.3 METs/min, moderate physical activity = 4.0 METs/min, and vigorous physical activity = 8.0 METs/min [83]. Total physical activity was calculated as the sum of METs per week. Patients were considered to be meeting the American Cancer Society guidelines [84] if they reported 600 or more MET minutes per week [85].

### 2.3. Data Analyses

Inflammatory biomarkers with estimates below the limit of detection were assigned a value of the limit of detection divided by the square root of 2. Indeterminate inflammatory biomarker concentrations were set to missing. Inflammatory biomarker values that were three standard deviations or more from the sample mean for each group were treated as outliers and set to missing. Raw inflammatory biomarkers with non-normal distributions (i.e., IL-6, IL-1ra) were natural-log-transformed to normalize their distributions, and the natural log values were used in analyses. To facilitate interpretation, both non-transformed data and transformed data are presented in selected tables. All cytokines that were not natural-log-transformed (i.e., IL-10, IL-1b, TNF-alpha, TNFR1, TNFR2, CRP) were mean-centered to facilitate interpretation.

Participants who provided a blood sample and questionnaire data at one or more timepoints were included in analyses. Means, standard deviations, frequencies, and percentages were used to describe sociodemographic and clinical characteristics of the sample. Differences in sociodemographic and clinical characteristics by group (i.e., patients vs. controls) were examined using independent sample t-tests, chi-square tests, and Fisher’s tests. Variables significant at *p* < 0.10 were included as covariates in longitudinal analyses. Inflammatory biomarkers and symptoms were described at each time point using means and standard deviations. Changes in inflammatory biomarkers and symptoms over time (for patients only and separately for controls only), and group differences (patients vs. controls) in these changes were evaluated using linear and quadratic random-effects mixed models and time coded as the number of months since baseline enrollment. When quadratic models were not significant, linear models were used instead. Cross-sectional group differences in inflammatory biomarkers and symptoms at each time point were examined using between-subject comparisons of least squares means from the quadratic random-effects mixed models, as these account for quadratic changes over time. Proportions of patients and controls with clinically meaningful fatigue, depression, problems with sleep, and physical inactivity were assessed at each timepoint.

Associations among inflammatory biomarkers and symptoms aggregated over the five assessments were evaluated using random-effects fluctuation mixed models [86]. Because of the time-varying nature of the inflammatory biomarkers, each model included both between-person predictors and within-person predictors. Between-person predictors tested whether participants’ average levels of each inflammatory biomarker differed from other participants’ average levels, and whether these differences were associated with symptoms. Within-person predictors tested whether participants’ average levels of each inflammatory biomarker differed from their own average level, and whether these differences were associated with symptoms. Interactions between fluctuations in inflammatory biomarkers and the group with symptoms were included in the fluctuation models to evaluate whether these associations between inflammation and symptoms were stronger for patients than controls. Significant interactions were further evaluated within each group using separate random-effects fluctuation models with the group effect and covariates removed. All statistical analyses were conducted using SAS version 9.4 (Cary, NC, USA).

## 3. Results

The sociodemographic and clinical characteristics of patients (*n* = 121) and controls (*n* = 105) are presented in Table 1. On average, patients were 60 years of age, married (70%), White (93%), and without a college education (63%). Most patients were diagnosed with stage 3 or 4 cancer (70%) of the ovaries or endometrium (75%). Controls were, on average, 58 years of age, White (89%), and college-educated (72%). Patients were less likely than controls to be college graduates (*p* < 0.01). Patients also reported more comorbidities (*p* = 0.04), on average, and were more likely to be post-menopausal than controls (*p* = 0.01). Age, education, comorbidities, and menopausal status were included as covariates in all multivariable analyses. Body mass index was not included, as it was not collected for controls. As expected, in this population where controls were age-matched to patients, there was not a statistically significant group difference in age. Nonetheless, age was included as a covariate because it is often associated with inflammation [87,88].

### 3.1. Group Differences in Inflammatory Biomarkers

**Figure 1:**
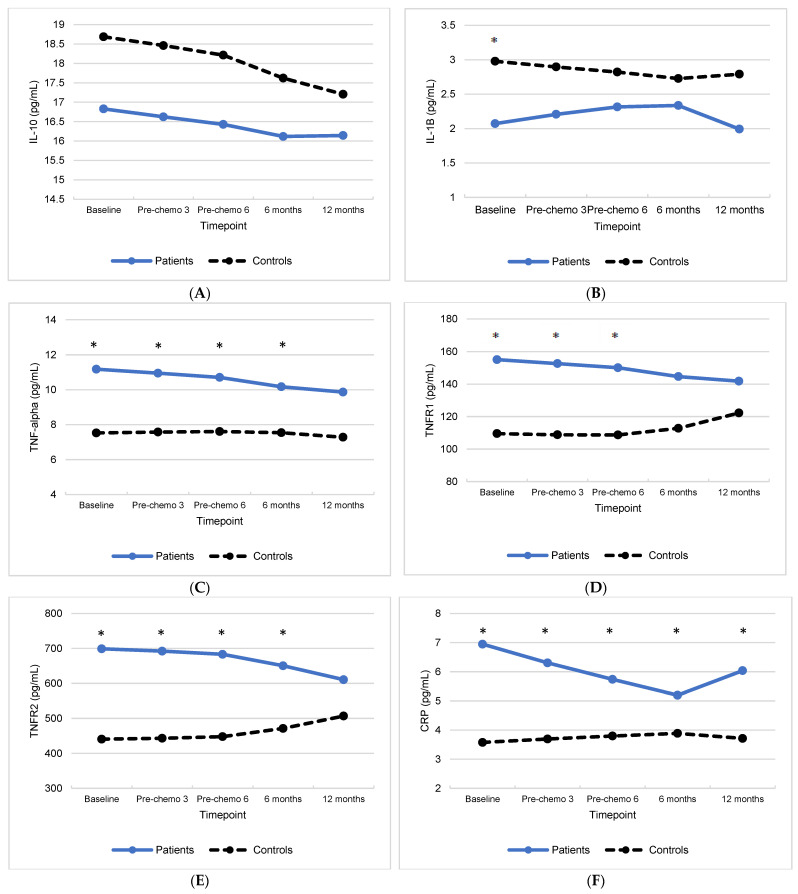

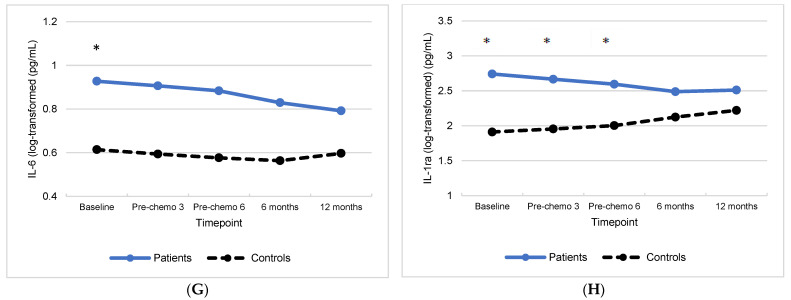
Least square mean levels over time for patients with gynecologic cancer and non-cancer controls for (**A**) IL-10, (**B**) IL-1B, (**C**) TNF-alpha, (**D**) TNFR1, (**E**) TNFR2, (**F**) CRP, (**G**) log-transformed IL-6, and (**H**) log-transformed IL-1ra. Asterisks indicate significant group differences in least squares means (*p* < 0.05).

Approximately 5% of inflammatory biomarker levels were below the lower limit of detection and divided by the square root of 2. Within each group, inflammatory biomarker levels that were more than three standard deviations from the group mean were set to missing (approximately 1% of all values, from 20 patients and 18 controls). Raw and log-transformed (IL-6 and IL-1ra only, per visual inspection of variable distributions) means for inflammatory biomarkers are displayed in Table 2. Adjusted means of circulating inflammatory biomarkers from the quadratic random-effects mixed models stratified by group are displayed in Figure 1A–H. Adjusted parameter estimates from the random-effects mixed models examining changes in inflammation over time are presented in Supplementary Table S1. Between-subject comparisons of adjusted means (i.e., least squares means) from the quadratic random-effects mixed models at each timepoint demonstrated that IL-1b was significantly lower in patients than controls at baseline only (*p* = 0.02). TNF-alpha and TNFR2 were significantly higher in patients than controls during active treatment (baseline, pre-chemo 3, and pre-chemo 6) and at the 6 month follow-up (*p*s < 0.04). TNFR1 and IL-1ra were significantly higher in patients than controls during active treatment (baseline, pre-chemo 3, and pre-chemo 6) (*p*s < 0.02). CRP was significantly higher in patients than controls at all timepoints (*p*s < 0.02). IL-6 was significantly higher in patients than controls at pre-chemo 3 only (*p* = 0.05).

Adjusted parameter estimates from the random-effects mixed models are presented in Supplementary Table S2. The results of the quadratic mixed models revealed a significant interaction between group and the quadratic effect of time for CRP (*p* < 0.001) such that, on average, levels of circulating CRP decreased from baseline to 6 months after completing chemotherapy and increased thereafter (*p* < 0.01) in patients, whereas controls’ levels of CRP remained unchanged over time (*p* = 0.29). The results of the linear mixed models revealed no significant changes in inflammation over time (*p*s > 0.05). There was a significant interaction between group and time for TNFR1 (*p* = 0.03), but the effect of time was not significant for patients or controls when each group was examined separately (*p*s > 0.05). There were also significant interactions between group and time for TNFR2 (*p* < 0.01) and IL-1ra (*p* = 0.03) such that levels of circulating TNFR2 and IL-1ra increased over time for controls (*p*s = 0.03) but not patients (*p*s > 0.05).

### 3.2. Group Differences in Treatment-Related Symptoms and Associations with Inflammation

**Figure 2:**
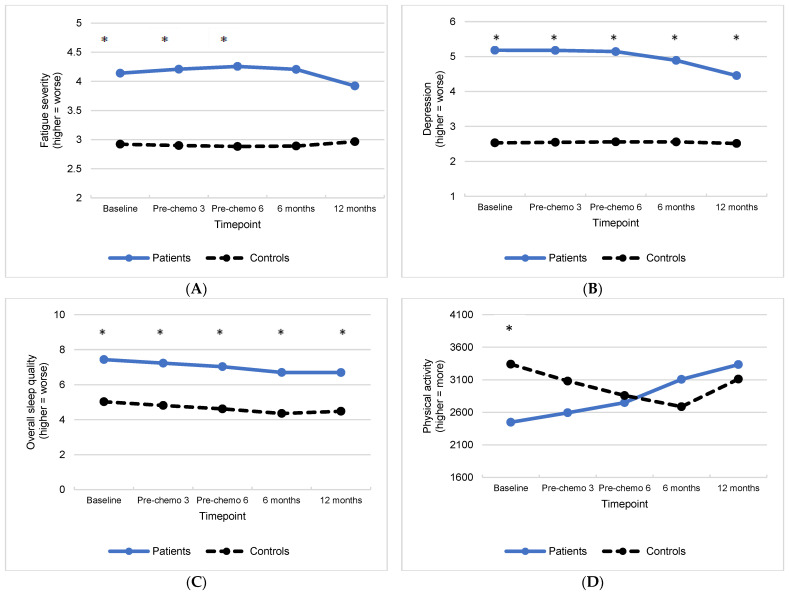
Least square mean levels over time for patients with gynecologic cancer and non-cancer controls for (**A**) fatigue severity (from the Fatigue Symptom Inventory), (**B**) depression (from the Hospital Anxiety and Depression Scale), (**C**) overall sleep quality (from the Pittsburgh Sleep Quality Index total score), and (**D**) total METS (from the International Physical Activity-Short Form). Asterisks indicate significant group differences in least squares means (*p* < 0.05).

The unadjusted mean levels of treatment-related symptoms (i.e., fatigue, depression, sleep, physical activity) over time are displayed in Table 2. The adjusted means of symptoms from the quadratic random-effects mixed models stratified by group are displayed in Figure 2A–D. The adjusted parameter estimates from the random-effects mixed models examining change in symptoms over time are presented in Supplementary Table S2. The results of the random-effects fluctuation models examining associations between inflammation and symptoms are presented in Supplementary Table S3.

#### 3.2.1. Fatigue

Between-subject comparisons of adjusted means at each timepoint revealed that patients reported significantly more fatigue than controls during active treatment (baseline, pre-chemo 3, and pre-chemo 6) (*p*s < 0.03). At baseline, 60% of patients reported clinically meaningful fatigue (i.e., Fatigue Severity Inventory scores of 3+), which increased to 72% by pre-chemo 6, compared to 39% and 36% of controls at the same timepoints. By 12 months after completing chemotherapy, 55% of patients and 45% of controls reported clinically meaningful fatigue. There was no significant change in fatigue over time, nor were there differences between groups in change in fatigue over time (*p*s > 0.05). There were no significant effects of circulating levels of inflammation or interactions between inflammation and group on fatigue (*p*s > 0.05).

#### 3.2.2. Depression

Patients reported significantly more depression than controls at all timepoints (*p*s < 0.01). At baseline, 23% of patients reported clinically meaningful depression (i.e., HADS depression scores of 8+), which increased slightly to 26% by pre-chemo 6 and decreased to 18% by 12 months after completing chemotherapy. In contrast, between 2% and 6% of controls reported clinically meaningful depression at any timepoint. Over time, there was no change in depression (*p* > 0.05), but there was a significant interaction between group and time (*p* = 0.05) such that depressive symptoms significantly improved over time in patients (*p* = 0.03) but remained unchanged for controls (*p* = 0.90). There were significant interactions of between-person variance in circulating TNF-alpha (*p* = 0.05) and IL-1ra (*p* = 0.04) with group on depression, but these effects were not significant for patients or controls when each group was examined separately (*p*s > 0.05). However, across groups, participants with higher circulating CRP levels had significantly greater depressive symptomatology (*p* = 0.01) than participants with lower CRP levels (i.e., main effect of between-person variance in circulating CRP). There were no other significant effects of circulating levels of inflammation or interactions between inflammation and group on depression.

#### 3.2.3. Sleep

Patients reported significantly worse overall sleep quality than controls at all timepoints (*p*s < 0.01). In total, 80% of patients reported clinically meaningful problems with sleep at baseline (i.e., PSQI total scores of 5+), which remained stable by pre-chemo 6 (78%) and decreased to 61% by 12 months after chemotherapy. In contrast, 50% of controls reported clinically meaningful problems with sleep at baseline, which remained stable by pre-chemo 6 (47%) and declined slightly to 41% at 12 months after chemotherapy. There was no significant change in sleep over time, nor were there differences between groups in change in sleep over time (*p*s > 0.05). There were no significant effects of circulating levels of inflammation or interactions between inflammation and group on sleep (*p*s > 0.05).

#### 3.2.4. Physical Activity

Patients reported significantly less physical activity than controls prior to chemotherapy only (*p* = 0.02). At baseline, 33% of patients did not meet the American Cancer Society guidelines for weekly METs expenditure (i.e., 600+ METs), compared to 11% of controls. By pre-chemo 6, 39% of patients reported under 600 METs per week, compared to 22% of controls, and 12 months after completing chemotherapy 21% of patients and controls reported under 600 METs per week. Over time, there was a main effect of group (*p* < 0.01) that was qualified by a significant interaction between group and time (*p* = 0.04) such that patients reported a linear increase in physical activity over time (*p* < 0.01), but controls’ physical activity decreased from baseline through the 6-month follow-up but increased thereafter (*p* < 0.01). There was a significant interaction of within-person fluctuations in circulating levels of TNFR1 with group on physical activity (*p* = 0.03), but this effect was not significant for patients or controls when each group was examined separately (*p*s > 0.05). Similarly, there was a significant interaction of between-person variance in circulating levels of IL-1ra by group on physical activity (*p* = 0.04), but this effect was not significant for patients or controls when each group was examined separately (*p*s > 0.05). However, at times when participant levels of circulating CRP were higher than their normal, they reported significantly less physical activity (*p* = 0.03) (i.e., main effect of within-person variance in circulating CRP).

## 4. Discussion

This study is among the first to examine relationships between circulating inflammatory biomarkers and treatment-related symptoms before, during, and after chemotherapy for gynecologic cancer. As hypothesized, inflammatory biomarkers were generally higher and fatigue, depression, and sleep disturbance were typically worse in patients with gynecologic cancer than controls. Physical activity was lower in patients before treatment and became more similar to controls over time. However, only CRP was associated with greater depression and less physical activity among both patients and controls. There were no associations with other inflammatory markers and fatigue or sleep disturbance.

Consistent with the previous literature [42,44,45], our results demonstrated that several inflammatory biomarkers (i.e., IL-1B, TNF-alpha, TNFR1, TNFR2, CRP, and IL-1ra) were elevated in patients prior to chemotherapy as compared to controls. The majority of these remained elevated in patients compared to controls before the third and sixth infusion of chemotherapy, and at 6 months and 12 months after completing chemotherapy, consistent with other studies of cancer patients [47,48,49,50,51,52]. For most of these cytokines (i.e., IL-10, IL-1b, TNFR2, or IL-6), there were no significant associations between symptoms and inflammation. In particular, between-person differences in inflammatory biomarkers were not associated with fatigue. While this finding is concordant with research on IL-6 and CRP in lung cancer patients undergoing chemotherapy [59] and in prostate cancer patients treated with androgen deprivation therapy [28], it is in contrast to research examining cytokines and fatigue in other cancer patient populations [30,58].

For patients only, there were quadratic changes in CRP levels over time such that CRP levels declined for patients only during chemotherapy, and subsequently increased by 12 months after treatment. Levels of CRP remained unchanged for controls assessed at similar timepoints. The results also indicated that both patients and controls with higher levels of circulating CRP than other participants, on average, reported worse depression. This is consistent with previous research identifying a significant association between CRP levels and depression severity in patients with other types of cancer [89,90,91,92]. In addition, women in our sample with higher levels of circulating CRP than their personal average reported less physical activity. This finding is consistent with prior research identifying higher CRP levels among post-treatment women with breast cancer who were less physically active than their personal average [93] and who exhibited poor physical fitness post treatment [94]. However, the associations between CRP and depression and physical activity were similar for patients and controls. Considering patients consistently reported worse symptomatology than controls, and patients demonstrated fluctuations in CRP over time that were not found in controls, one possible explanation for the lack of a group-based difference in the association between CRP and depression is that there are multiple triggering factors of symptoms, such as oxidative stress, genetic risk factors, metabolic dysregulation, or host and microbiome genomic risk factors [41,95]. For patients, symptoms may also be perpetuated by other symptoms, irrespective of systemic inflammation [41].

This study had several strengths, including a longitudinal study design, the inclusion of on-treatment and post-treatment pro-inflammatory cytokine and symptom data, and a non-cancer comparison group. However, study limitations should also be noted. The sample was relatively homogeneous in terms of race and ethnicity, which could limit the generalizability of these findings. We did not collect height and weight data from controls; thus, we could not include body mass index as a covariate in longitudinal analyses. We also had limited statistical power to identify moderate associations between fluctuations in inflammatory biomarkers and symptoms throughout the treatment period. Of note, some patients may have been taking anti-inflammatory drugs (e.g., NSAIDS); thus, we may have underestimated inflammation levels. Additional research is needed to replicate these findings in larger and more diverse samples.

## 5. Conclusions

In conclusion, our results suggest that several inflammatory biomarkers and symptoms are elevated or worse in gynecologic cancer patients prior to, during, and after chemotherapy compared to non-cancer controls. For both patients and controls, levels of CRP were associated with worse symptomatology (i.e., depression and physical activity). These associations were no stronger for patients than for controls, suggesting that there may be other causal mechanisms of symptoms in gynecologic cancer patients beyond inflammation. Additional research is needed to identify additional biological mechanisms of symptoms and examine whether reductions in inflammation (e.g., circulating levels of CRP) can reduce the severity of symptoms in patients treated with chemotherapy. Such interventions may considerably improve the quality of life of patients with gynecologic cancer treated with chemotherapy. Further, reductions in inflammation may improve quality of life in individuals without cancer.

## Figures and Tables

**Table 1:** Participant characteristics of patients with a diagnosis of gynecologic cancer and non-cancer controls.

Variable	Patients (*n* = 121)	Controls (*n* = 105)	*p*-Values
Age: M (SD)	60.4 (10.7)	58.0 (12.8)	0.12
Race: *n* (%) White	112 (93)	93 (89)	0.11
Education: *n* (%) college graduate	45 (37)	76 (72)	<0.0001
Income: *n* (%) USD 40 k or more	66 (69)	74 (80)	0.08
Comorbidities: mean (SD) [range]	2.4 (0.8) [2–7]	2.2 (0.5) [2–5]	0.04
Menopausal status: *n* (%)			0.01
Pre-menopausal	15 (13)	27 (26)	
Post-menopausal	104 (87)	78 (74)	
Cancer type: *n* (%)		-	-
Cervical	4 (3)		
Endometrial	27 (22)		
Fallopian	5 (4)		
Ovarian	64 (53)		
Peritoneal	7 (6)		
Uterine	10 (8)		
Vulvar	1 (1)		
Other	2 (2)		
Stage: *n* (%)		-	-
1	22 (19)		
2	13 (11)		
3	61 (54)		
4	18 (16)		
Prior lines of chemotherapy: *n* (%) 3 or more	14 (12)	-	-

**Table 2:** Mean scores for biomarkers of inflammation and treatment-related symptoms over time for patients with gynecologic cancer and non-cancer controls.

	Baseline		Pre-Chemotherapy Cycle 3		Pre-Chemotherapy Cycle 6		6-Month Follow-Up		12-Month Follow-Up	
	Patients Mean (SD)	Controls Mean (SD)	Patients Mean (SD)	Controls Mean (SD)	Patients Mean (SD)	Controls Mean (SD)	Patients Mean (SD)	Controls Mean (SD)	Patients Mean (SD)	Controls Mean (SD)
Biomarkers of Inflammation (pg/mL):										
IL-10	24.4 (21.1)	23.0 (16.7)	22.3 (18.4)	23.3 (14.8)	22.7 (18.0)	24.2 (14.8)	23.7 (18.5)	22.7 (15.9)	25.7 (20.1)	23.2 (15.6)
IL-1b	3.4 (2.5)	3.9 (2.9)	3.4 (2.6)	4.0 (2.7)	3.5 (2.6)	4.1 (2.8)	3.4 (2.1)	4.0 (2.8)	3.4 (2.6)	4.4 (3.2)
TNF-alpha	16.3 (7.9)	12.0 (6.6)	15.4 (7.9)	12.3 (5.7)	15.8 (7.4)	12.4 (7.3)	16.0 (7.1)	12.6 (6.8)	16.6 (7.5)	12.7 (6.3)
TNFR1	181.6 (107.7)	126.0 (78.4)	178.7 (106.5)	126.1 (82.8)	185.3 (105.9)	133.7 (90.7)	187.9 (105.1)	132.6 (74.9)	185.4 (100.1)	133.5 (80.5)
TNFR2	1015.4 (580.2)	733.2 (313.8)	1078.3 (626.3)	748.3 (363.6)	1071.7 (560.6)	757.5 (326.5)	1077.0 (581.1)	793.9 (348.0)	1021.3 (474.4)	758.5 (334.4)
CRP	6.2 (4.6)	2.9 (2.9)	6.2 (4.7)	3.0 (3.1)	5.6 (4.9)	3.0 (2.8)	5.0 (4.6)	2.9 (2.8)	5.4 (5.1)	2.5 (2.2)
IL-6	6.4 (4.8)	4.4 (3.0)	5.6 (3.5)	4.5 (2.7)	5.5 (3.4)	4.5 (2.8)	6.3 (5.2)	4.6 (2.8)	5.7 (4.3)	4.4 (2.8)
IL-6 (log- transformed)	1.5 (1.1)	1.0 (1.4)	1.4 (1.2)	1.2 (1.2)	1.4 (0.9)	1.1 (1.4)	1.4 (1.4)	1.1 (1.4)	1.5 (0.9)	1.0 (1.4)
IL-1ra	62.9 (94.2)	55.1 (101.5)	59.6 (84.6)	59.7 (103.7)	49.9 (80.1)	50.3 (97.2)	74.5 (132.1)	59.3 (118.9)	93.8 (139.1)	54.4 (100.8)
IL-1ra (log- transformed)	3.4 (1.4)	2.5 (1.8)	3.2 (1.4)	2.6 (1.8)	3.0 (1.4)	2.3 (1.8)	3.3 (1.5)	2.5 (1.8)	3.4 (1.9)	2.4 (1.8)
Sickness Behaviors:										
Fatigue ^1^	3.4 (1.9)	2.5 (1.6)	3.7 (1.7)	2.6 (1.8)	4.3 (2.2)	2.4 (1.7)	3.4 (2.0)	2.4 (1.9)	3.2 (2.0)	2.6 (1.9)
Depression ^2^	4.7 (3.7)	2.1 (2.2)	5.0 (3.9)	2.2 (2.7)	5.6 (3.9)	2.1 (2.6)	3.9 (3.4)	2.1 (2.5)	3.9 (3.4)	2.2 (2.4)
Overall Sleep Quality ^3^	7.2 (3.5)	4.9 (2.8)	7.4 (4.0)	4.9 (2.7)	6.8 (3.4)	5.2 (3.0)	6.9 (3.8)	4.1 (2.9)	6.5 (4.2)	4.7 (3.1)
Physical Activity (Total METs) ^4^	1771 (2254)	2744 (2440)	1984 (2205)	2700 (2825)	2003 (3281)	2408 (2783)	2590 (3051)	2455 (2808)	2618 (3155)	2336 (2639)

Note: IL-10 = interleukin-10; IL-1b = interleukin 1 beta; TNF-alpha = tumor necrosis factor–alpha; TNFR1 = tumor necrosis factor receptor 1; TNFR2 = tumor necrosis factor receptor 2; CRP = c-reactive protein; IL-6 = interleukin-6; IL-1ra = interleukin 1 receptor antagonist. ^1^ Measured using the Fatigue Symptom Inventory; scores of 3 or above indicate clinically meaningful fatigue. ^2^ Measured using the Depression subscale of the Hospital Anxiety and Depression Scale; scores of 8 or higher indicate clinically meaningful depressive symptoms. ^3^ Measured using the total score from the Pittsburgh Sleep Quality Index; scores of 5 and above indicate clinically meaningful sleep problems. ^4^ Measured using the International Physical Activity Questionnaire-Short Form; fewer than 600 MET-minutes per week indicated non-adherence to American Cancer Society physical activity guidelines.

## Data Availability

The data presented in this study can be shared upon reasonable request.

## Supplementary Materials

The following supporting information can be downloaded at: https://www.mdpi.com/article/10.3390/cancers15133407/s1, Table S1. Adjusted parameter estimates from mixed models examining group differences in changes in biomarkers of inflammation among patients with gynecologic cancer treated with chemotherapy and non-cancer controls; Table S2. Adjusted parameter estimates from mixed models examining group differences in changes in treatment-related symptoms among patients with gynecologic cancer treated with chemotherapy and non-cancer controls; Table S3. Adjusted parameter estimates from mixed models examining associations between fluctuations in biomarkers of inflammation with symptoms among patients with gynecologic cancer treated with chemotherapy and non-cancer controls.
