# Supplementary material for: Relationships among Inflammatory Biomarkers and Self-Reported Treatment-Related Symptoms in Patients Treated with Chemotherapy for Gynecologic Cancer: A Controlled Comparison
Source: Cancers (Basel). 2023 Jun 29;15(13):3407. doi: 10.3390/cancers15133407 (PMC10340589; doi:10.3390/cancers15133407)
Supplement: Supplementary file 1 [file cancers-15-03407-s001.zip › cancers-2426052-supplementary.pdf]

Supplemental Table S1. Adjusted parameter estimates from mixed models examining group differences in changes in biomarkers of inflammation among patients with gynecologic cancer treated with chemotherapy and non-cancer controls

	IL-10	IL-1b	TNF-alpha	TNFR1	TNFR2	CRP	IL-6^	IL-1ra^
Intercept	21.84**	2.85**	6.77*	102.98*	403.24*	3.58*	0.77	1.69**
Group	-2.51	-0.76*	3.26***	43.39**	256.07***	3.37***	0.35*	0.74**
Time	-0.09	-0.01	-0.01	0.70	3.81	0.06	-1.86E-03	0.02
Group*Time	0.05	7.27E-03	-0.07	-1.48*	-9.03**	-0.42***	-6.10E-03	-0.03*
Time*Time	-	-	-	-	-	-3.40E-03	-	-
Group*Time*Time	-	-	-	-	-	0.02***	-	-

Note: Analyses controlled for age, education, comorbidities, and menopausal status.
^ IL-6 and IL-1ra were natural log-transformed. A main effect of group indicates group differences at the intercept (i.e., pre-chemotherapy baseline). An interaction between group and time indicates differences in changes in biomarkers of inflammation by treatment group (i.e., patients vs. non-cancer controls). When there was not a significant quadratic effect, linear estimates are presented.
*** = $p<0.001$; ** = $p<0.01$; * = $p<0.05$

Supplemental Table S2. Adjusted parameter estimates from mixed models examining group differences in changes in treatment-related symptoms among patients with gynecologic cancer treated with chemotherapy and non-cancer controls

	Fatigue	Depression	Overall Sleep Quality	Physical Activity
Intercept	2.76***	2.27*	5.26***	3630.03
Group	1.26***	2.71***	2.47***	-898.82*
Time	3.13E-03	-1.33E-03	-0.03	142.32**
Group*Time	-0.02	-0.04*	-0.01	217.33**
Time*Time	-	-	-	7.57**
Group*Time*Time	-	-	-	-8.90*

Note: Analyses controlled for age, education, comorbidities, and menopausal status. A main effect of group indicates group differences at the intercept (i.e., pre-chemotherapy baseline). An interaction between group and time indicates differences in changes in symptoms by treatment group (i.e., patients vs. non-cancer controls). When there was not a significant quadratic effect, linear estimates are presented.

*** = p<0.001; ** = p<0.01; * = p<0.05

Supplemental Table S3. Adjusted parameter estimates from mixed models examining associations between fluctuations in biomarkers of inflammation with symptoms among patients with gynecologic cancer treated with chemotherapy and non-cancer controls

	Fatigue Severity							
	IL-10	IL-1b	TNF-alpha	TNFR1	TNFR2	CRP	IL6^	IL-1ra^
Intercept	2.60***	3.00***	2.86***	2.65***	2.51**	2.71***	2.98***	3.05***
Group	1.52***	1.14**	1.11*	1.34**	1.62**	1.19**	1.18***	1.15**
Between-person cytokine	0.02	-0.01	0.02	2.28E-03	4.98E-04	0.09	0.04	-0.07
Within-person cytokine	-0.02	-0.02	-0.03	2.08E-03	3.92E-04	-2.80E-04	-0.03	0.03
Group x between-person cytokine	-0.01	0.01	2.90E-04	-1.28E-03	-4.80E-04	-4.05E-02	-0.08	0.09
Group x within-person cytokine	7.33E-03	-0.03	0.03	-5.20E-04	1.40E-05	-3.03E-03	0.01	-0.11

	Depression							
Variable	IL-10	IL-1b	TNF-alpha	TNFR1	TNFR2	CRP	IL6^	IL-1ra^
Intercept	2.07	2.74*	2.85*	1.91	1.32	1.18	2.29	2.84*
Group	2.11**	2.11**	0.88	2.78**	3.50***	2.63***	2.28***	1.56*
Between-person cytokine	-9.22E-03	-0.13	-0.05	2.02E-03	1.23E-03	0.27*	-0.23	-0.23
Within-person cytokine	-9.16E-03	-8.11E-03	6.59E-03	-1.17E-03	-1.20E-04	4.95E-04	-0.07	-0.01
Group x between-person cytokine	0.03	0.13	0.12*	-1.00E-03	-1.08E-03	-0.13	0.60	0.59*
Group x within-person cytokine	-0.01	1.68E-03	5.27E-03	1.72E-03	7.88E-04	0.04	0.19	-0.07

	Overall Sleep Quality							
Variable	IL-10	IL-1b	TNF-alpha	TNFR1	TNFR2	CRP	IL6^	IL-1ra^
Intercept	5.56***	5.36***	4.68***	5.51***	4.81***	5.25***	5.42***	5.72***
Group	2.28**	2.08***	3.94***	2.88**	4.13***	2.13**	2.30***	3.01***
Between-person cytokine	-5.84E-03	-0.02	0.08	1.84E-04	7.70E-04	0.10	-0.13	-0.24
Within-person cytokine	-0.02	0.05	-1.48E-03	8.85E-04	2.26E-04	0.10	0.05	-0.15
Group x between-person cytokine	9.89E-03	-0.18	-0.11	-2.53E-03	-1.75E-03	0.03	-0.03	-0.28
Group x within-person cytokine	0.02	-0.15	0.06	4.86E-03	6.76E-04	0.10	0.15	0.17

	Total METS							
Variable	IL-10	IL-1b	TNF-alpha	TNFR1	TNFR2	CRP	IL6^	IL-1ra^
Intercept	3128.75**	3506.85***	2934.28**	3193.57**	3871.27***	3941.09***	3040.65**	3363.51***
Group	-68.91	-829.62	-482.03	-585.85	-1656.34*	-979.92	-70.86	-1186.30*
Between-person cytokine	2.35	112.69	31.99	0.36	-0.62	-159.65	-16.13	-206.74
Within-person cytokine	14.67	-38.37	-31.53	1.18	0.14	-142.79*	-25.54	7.00
Group x between-person cytokine	-15.32	115.43	-2.56	0.81	1.30	176.37	-134.35	460.69*
Group x within-person cytokine	-21.27	70.13	-9.42	-8.42*	-1.28	30.72	-215.11	-71.09

Note: Analyses controlled for age, education, comorbidities, and menopausal status.

^ IL-6 and IL-1ra were natural log-transformed.

*** = p<0.001; ** = p<0.01; * = p<0.05
